# From Origins to Trends: A Bibliometric Examination of Ethical Food Consumption

**DOI:** 10.3390/foods13132048

**Published:** 2024-06-27

**Authors:** Silviu Beciu, Georgiana Armenița Arghiroiu, Maria Bobeică

**Affiliations:** Faculty of Management and Rural Development, University of Agricultural Sciences and Veterinary Medicine of Bucharest, 59 Marasti, Sector 1, 011464 Bucharest, Romania; beciu.silviu@managusamv.ro (S.B.); armenitaarghiroiu@gmail.com (G.A.A.)

**Keywords:** ethical food consumption, bibliometric analysis, consumer behavior, sustainability, food ethics

## Abstract

Ethical food consumption has gained significant attention in the past years, reflecting a societal shift towards ethical behavior. Our study examines the evolution of ethical food consumption research over the past three decades, aiming to map its transformation. We identified key trends, influential contributors, and major thematic clusters through a bibliometric analysis, employing VOSviewer (v.1.6.18) for bibliometric visualization, focusing on citation networks and keyword o-occurrences to reveal the field’s structure and dynamics. We made extensive use of the Web of Science database, where we selected 1096 relevant articles and review papers. Our analysis shows a notable rise in publications starting in 2005, with a peak in 2022, indicating increased scholarly interest in the topic. The findings underscore the importance of integrating empathy and human values into ethical food consumption, highlighting the critical roles of animal welfare, sustainability, and social justice. Despite a strong pro-ethical attitude among consumers, a significant “attitude-behavior gap” persists, emphasizing the need for strategies that bridge this divide. Our results emphasize the importance of interdisciplinary efforts to align ethical practices with broader societal goals, offering valuable insights for future research and policy-making to promote sustainable and ethical food consumption worldwide.

## 1. Introduction

The concept of ethical food consumption covers an extensive range of principles and actions directed at addressing the moral aspects of how food is produced, distributed, consumed, and disposed of [1,2]. In academic engagements, ethical eating involves consumers deliberately choosing food options they believe will positively impact the food system, such as selecting organic, plant-based, fair trade, or locally sourced products [3]. However, the definitions and practices associated with ethical eating are often seen as incomplete, especially because ethical consumption alone cannot fully resolve complex issues like access to resources, local food prevalence, and efficient production [4,5,6].

Over the past few decades, there has been a growing awareness of consumption sustainability [7] and the ethical implications of our dietary choices [8], with increasing emphasis on sustainable practices [9], animal welfare [10], and social justice [11]. As consumers become more conscious of these issues, the academic community has responded with a generous body of research aimed at understanding and promoting ethical food practices.

Public policies play a fundamental role in shaping the widespread dynamics of the discourse on ethical food consumption. The term itself is ambivalent, utilized either by civil society movements advocating for systemic changes in the food system or by corporations that see it as a way to exploit niche markets or popular consumption trends [12]. Conversely, the popularity of such concepts is more often than not directly influenced by societal development, with privileged populations often having easier access to prevalent ethical eating practices while less developed societies tend to focus more on survival than on idealistic concepts [13]. There is evidence that ethical consumption can have status implications, especially when combined with aesthetic food preferences, suggesting that the most privileged consumers often integrate both ethical and aesthetic dimensions into their food choices [14].

While numerous studies developed on the relationship between ethics and food-consuming habits, we noticed that review papers that treat the subject as a whole are relatively scarce. Most papers tend to focus on specific aspects of ethical food consumption, such as consumption experiences [15,16], environmental care [17,18,19,20], alternatives to traditional protein sources [21,22,23,24,25] or health [26,27]. Recognizing this gap, we decided to conduct a bibliometric study that takes a multi-topic approach, analyzing all papers related to ethical food consumption to provide a holistic bibliometric review. Despite the growing body of literature on ethical food consumption, there also is a lack of inclusive analysis on the evolution of empathy and its role in shaping ethical food choices over time. Ethical food consumption becomes closely connected to consumer identity, encompassing concepts like care, altruism, and recognition of the challenges and suffering involved in food production and consumption [28]

Understanding the long-term role of empathy in the consumer decision-making process can offer valuable insights for policymakers, educators, and marketers, promoting more sustainable and humane food practices.

While we acknowledge the wide-reaching ramifications of the present topic, our purpose is to focus on exploring several pertinent questions within the sphere of ethical food consumption:

Q1. What trends have emerged in the scientific research on ethical food consumption over time?

Q2. Who are the most influential authors, publications, countries, and research themes in the field of ethical food consumption, and how have they shaped current discourse and practices?

Q3. What are the key thematic clusters and research trends in the literature on ethical food consumption, as revealed through bibliometric analysis?

Q4. What is the relative prevalence of practical approaches versus theoretical models in researching ethical food consumption?

Q5. What emerging ethical issues in the global food chain are likely to gain traction due to increasing consumer empathy, and how can future research address these issues?

The primary objective of this bibliometric analysis is to provide a detailed overview of the current literature on ethical food consumption. Specifically, this study aims to:

▪ Identify emerging trends in the scientific research on ethical food consumption.
▪ Determine the most influential authors, publications, countries, and research themes in the field.
▪ Analyze key thematic clusters and research trends through bibliometric techniques.
▪ Compare the prevalence of practical approaches versus theoretical models in the literature.
▪ Identify emerging ethical issues in the global food chain likely to gain traction due to increasing consumer empathy.

Continuing, this paper is structured as follows: Section 2 details the methodology used in the study. Section 3 presents the results of the bibliometric analysis, highlighting key trends, influential works, and thematic clusters, discussing the implications of the findings, and identifying gaps in the literature. Finally, Section 4 concludes the paper with recommendations for future research and practical applications.

## 2. Materials and Methods

We aimed to provide an in-depth analysis of the existing literature on ethical food consumption, referencing papers by Donthu N. [29] and Ozturk [30].

In selecting our data source, we evaluated databases known for their extensive article collections, including Web of Science, Scopus, Google Scholar, and JSTOR. We opted for Web of Science due to its wide-ranging coverage across academic disciplines and its compatibility with the VOSviewer visualization tool. After careful consideration of alternative analysis and visualization tools such as Leximancer, CiteSpace, and Gephi, we chose VOSviewer for its visual capabilities, which are more suited to the objectives of our study.

After previously researching the concepts of food ethics and consumer behavior, we focused on finding relevant articles and review papers for the “Ethical Food Consumption” Web of Science search, performing our search in April 2024. Articles were selected if they focused on ethical food consumption, were peer-reviewed, and explicitly addressed ethical considerations related to our topic, such as sustainability, fair trade, or animal welfare. Our search also focused only on English language papers. We excluded articles that did not explicitly address our research questions or lacked relevance to the main themes of our study. Also, we removed any duplicates or irrelevant entries in the dataset. The final list of papers included in our analysis consisted of 986 articles and 110 review articles that met the inclusion/exclusion criteria.

For our next step, we extracted relevant bibliographic information from the selected articles, including publication titles, authors, publication years, journal names, and citation counts. This information formed the basis for our bibliometric analysis, allowing us to examine publication trends, citation networks, and thematic clusters within the literature on ethical food consumption. While VOSviewer (v.1.6.18) was our tool for the generation of comprehensive visual maps, we also used Microsoft Excel for graphs and charts which required a more succinct approach.

Subsequently, the extracted data were imported into VOSviewer (v.1.6.18) software, which facilitates the creation of network maps, enabling the exploration of citation patterns, co-citation relationships, and keyword co-occurrence within the dataset. Citation analysis maps provided insights into the flow of scholarly influence among publications, while co-citation analysis maps identified clusters of closely related articles based on shared citations.

The results helped us understand how knowledge is shared, how different research themes are connected, and which contributions are most important in the field of ethical food consumption. We identified key areas of research, top authors, and important publications in the field.

Overall, our methodology aimed to provide a rigorous and systematic approach to analyzing the scholarly literature on ethical food consumption, utilizing bibliometric techniques to uncover trends, patterns, and dynamics within this evolving field of study.

## 3. Results and Discussion

Table 1 contains a concise summary of the data sourced from Web of Science, highlighting the initial dimension of the dataset to be analyzed:

*Q1. What trends have emerged in the scientific research on ethical food consumption over time?*

While more consistent scientific literature addressing ethical food consumption has been published fairly recently, this topic is not new. For a long time, people have thought about whether it is right or wrong to eat a certain way. This includes thinking about it from a philosophical perspective or considering what religions say about their way of living. From ancient Greek times up to the present day [31], debates have persisted regarding the moral integrity of our consumption behaviors, from what we eat to what we wear or otherwise use in our day-to-day lives.

The oldest article that appeared in our search results is from 1991, “Organoleptic Examination of Food” [32]. This paper mentions ethical food consumption by referencing much older German materials, such as *The German Food Code*, also known as *Deutsches Lebensmittelbuch*, which was first sketched in 1962 because authorities identified a need to establish standards for the composition, ethical production, and appropriate marketing of food, while also meeting consumer expectations [33].

**Figure 1:**
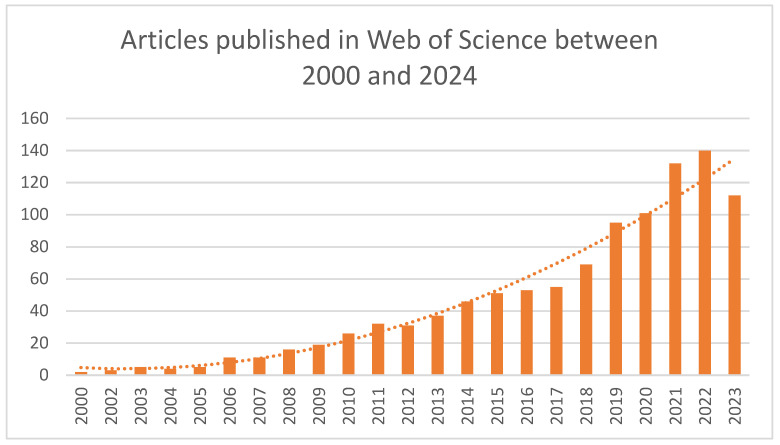
Evolution of articles number between 2000 and 2024—data from Web of Science database.

After this, interest in this topic rose relatively slowly, with no more than five articles per year until 2005. After 2005 the topic was more actively explored, reaching its peak in 2022, with 140 articles published. More than 80% of the total articles mentioning ethical food consumption available on Web of Science were published in the past 10 years (Figure 1). Sterie and team [34] identified a similar trend in their review paper, highlighting studies related to the Fair Food Trade aspect of sustainable food consumption.

**Figure 2:**
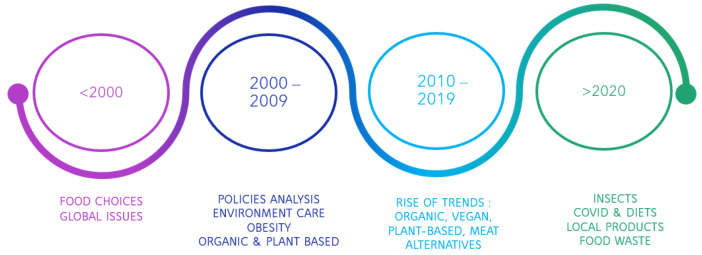
Recurring subjects, by decade—data from the Web of Science database.

We have observed a shift in the focus of research on ethical food consumption over different decades (Figure 2). Before the year 2000, the approach was predominantly theoretical, encompassing broader themes such as globalization, health considerations, and the societal impacts of dietary choices. Over time we saw more and more focalized materials, and as we transitioned into the third millennium, there was a notable surge in scholarly output addressing more diverse ideas and topics. In 2005, we found one of the first reviews that tackled the subject. This review addressed the issue of ethical food consumption by emphasizing the need for more efficient resource utilization in food production, as well as the need to ensure access to safe drinking water while producing food, in the face of water scarcity [35,36].

The first decade of the 21st century also witnessed research focusing on policy analysis and environmental impacts related to ethical food consumption. Moreover, there was a growing concern for diets aimed at combating obesity [37,38], as well as an increasing interest in the ethical dimensions of organic and plant-based foods [39,40,41,42].

The decade between 2010 and 2019 can be marked as the “trends” decade. We observe more and more articles focused on different dietary patterns and the level of fairness in consuming them [43,44]. Meat alternatives are increasingly studied, driven in part by ethical considerations regarding animal welfare [45,46,47].

The onset of the COVID-19 pandemic in early 2020 prompted a shift in focus towards promoting healthy and ethical food choices, including an emphasis on buying locally produced goods and shortening food supply chains [48]. The pandemic disrupted long food supply chains, creating an opportunity for local farmers to step in and provide solutions through shorter and more sustainable food chains [49]. Additionally, there was a renewed interest in advocating for insect-based proteins as viable substitutes for traditional meat consumption [50,51,52].

As we move towards the second half of the current decade, we observe a growing interest in exploring alternative food sources such as algae and genetically engineered/modified foods, alongside a continued trend toward reducing meat consumption.

*Q2. Who are the most influential authors, publications, countries, and research themes in the field of ethical food consumption, and how have they shaped current discourse and practices?*

**Figure 3:**
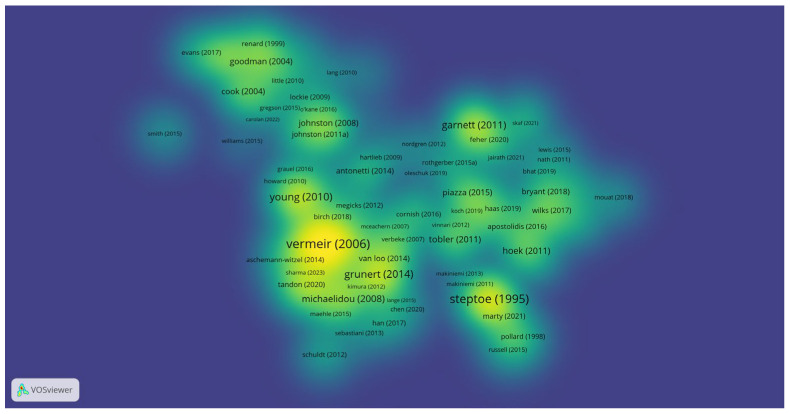
VOSviewer Density visualization of most cited papers on ethical food consumption, representing 22 clusters. N = 376 (documents with a minimum of 20 citations per document).

In examining the most cited documents within our dataset, we set a threshold of a minimum of 20 citations per document, resulting in the identification of 376 articles meeting this criterion out of a total of 1096 articles (Figure 3).

Among the most cited papers, notable contributions include Vermeir’s work from 2006 [53] which underscores the significance of accessible information in shaping sustainable consumption behaviors. Steptoe’s study from 1995 [54] investigates the influence of personal moral convictions on ethical behavior, while Young’s research from 2010 [55] delves into the consumer journey of green purchasing and highlights the barriers between intention and action. Notably, each of these papers has garnered over 500 citations, indicating their substantial impact within the field.

Additionally, research clusters have emerged around influential authors such as Johnston [15] Goodman [56], and Garnett [57] suggesting the prominence of their respective contributions to the discourse on ethical food consumption. These scholars have likely played pivotal roles in shaping research trajectories and advancing understanding within this domain.

**Figure 4:**
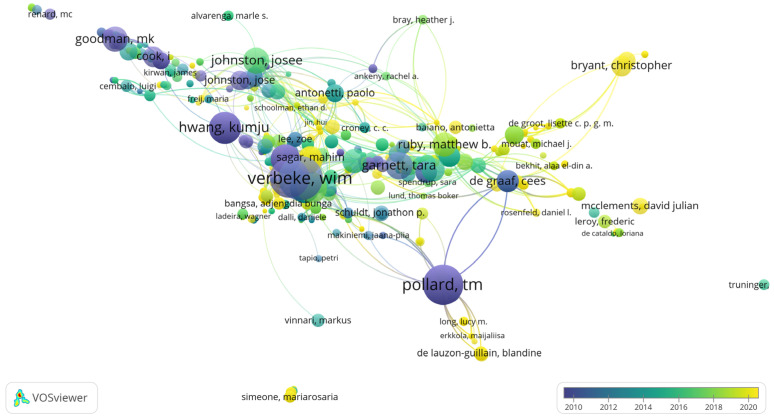
VOSviewer Overlay visualization of most cited authors, representing 26 clusters. Included 760 sources with a minimum of 20 citations per source.

In examining documents by author, we established a minimum threshold of at least one document per author with a minimum of 20 citations per author. Out of 3353 authors, 1160 met these criteria (Figure 4).

Among these authors, Verbeke Wim emerges as particularly prominent in terms of citations. His work approaches ethical consumption through the lens of fair animal treatment and meat consumption. Furthermore, Verbeke’s research delves into the marketing approach to ethical eating, providing valuable insights into the complex nature of ethical food consumption and its societal implications (Table 2). His paper “Sustainable food consumption: Exploring the consumer attitude—behavioral intention gap” is also the most cited in our list. Overall, Verbeke gathers a total of 1943 citations, being the most cited author in the current dataset.

**Figure 5:**
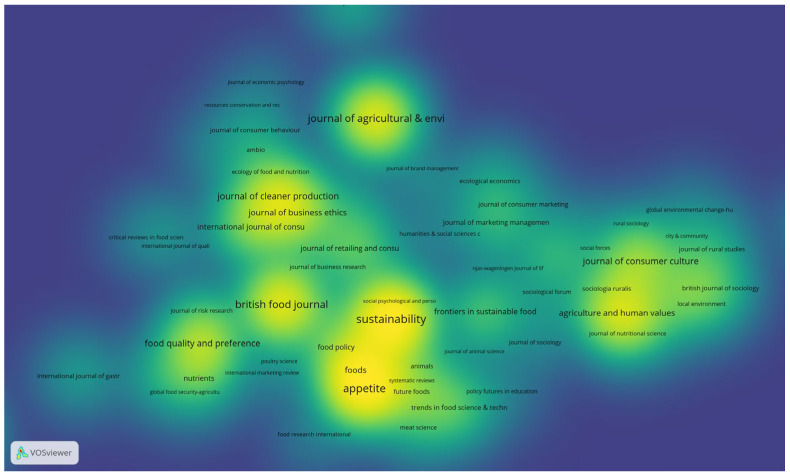
VOSviewer Density visualization of most cited sources, representing 20 clusters. Included 187 sources with a minimum of 20 citations per source.

In scrutinizing documents according to their source, we set a threshold requiring at least one document per source with a minimum of 20 citations per source. Out of 523 sources, 187 met these criteria (Figure 5). Notably, the top three most cited journals were identified as *Appetite* (with 4735 citations), *Journal of Agricultural and Environmental Ethics* (with 2291 citations), and *Food Policy* (with 1970 citations). Furthermore, among the 13 sources surpassing a total of 500 citations, these journals are prominent players in disseminating research on ethical food consumption, reflecting their significant influence within the academic community (Table 3).

**Figure 6:**
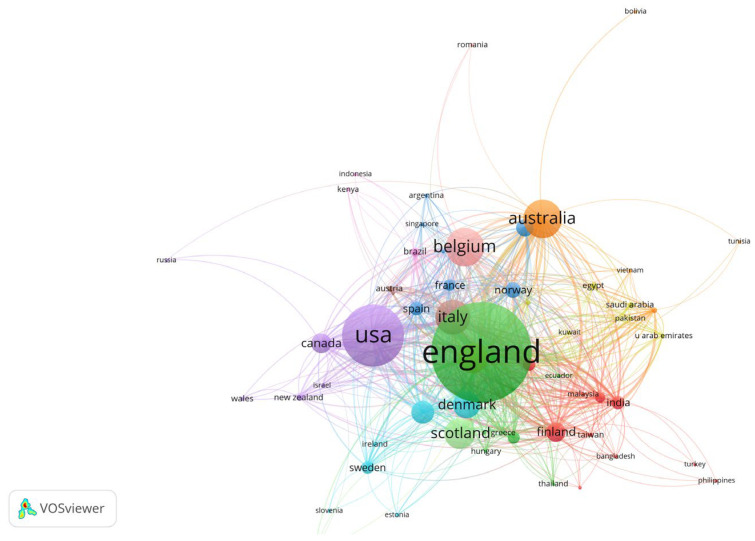
VOSviewer Network visualization of most cited countries, representing 11 clusters. Included 68 countries with a minimum of 20 citations per source.

In the analysis of documents according to their country of origin, we established a threshold requiring at least one document per country with a minimum of 20 citations per source. Out of 92 countries, 68 met these criteria (Figure 6). The widespread participation of numerous countries in researching this topic underscores the considerable interest and significance attached to ethical food consumption within the global discourse. The most prominent countries that are the origin of highly cited papers are England, the USA, Belgium, and Australia. Seven out of the first ten countries are from Europe.

**Figure 7:**
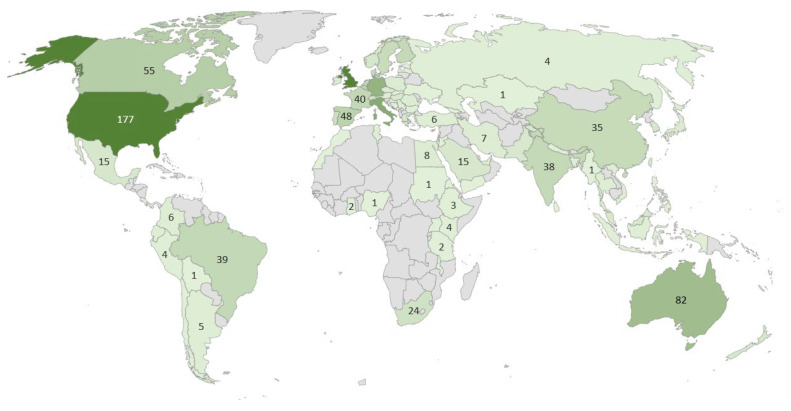
Number of articles per country, Web of Science data.

However, upon examining the map generated from the total number of articles by country of origin (Figure 7), it becomes apparent that more developed societies tend to prioritize the issue of ethics in food consumption research, while underdeveloped or developing countries, particularly those in Africa, parts of Asia, and South America, exhibit minimal to no representation. This disparity in geographical distribution highlights potential inequalities in research focus and resource allocation within the realm of ethical food consumption, calling attention to the need for greater inclusivity and collaboration across diverse socio-economic contexts.

Research topics are often closely tied to regional concerns and the specific socio-economic conditions of the countries. For instance, while advanced economies like the USA and Australia focus extensively on sustainability and animal welfare, researching alternatives to traditional food chains [63,64], and European countries focus on sustainable gastronomy [65], countries like Ethiopia [66] and Uganda [67] are more concerned with basic public health and nutritional diversity. Similarly, Tunisia’s unique focus on resistance to modern retailing [68] highlights the cultural dimensions of ethical consumption, which are not as prominently discussed in other regions. In examining when ethical consumption becomes a high-status practice, it becomes clear that societal values and economic status significantly influence food preferences.

*Q3. What are the key thematic clusters and research trends in the literature on ethical food consumption, as revealed through bibliometric analysis?*

**Figure 8:**
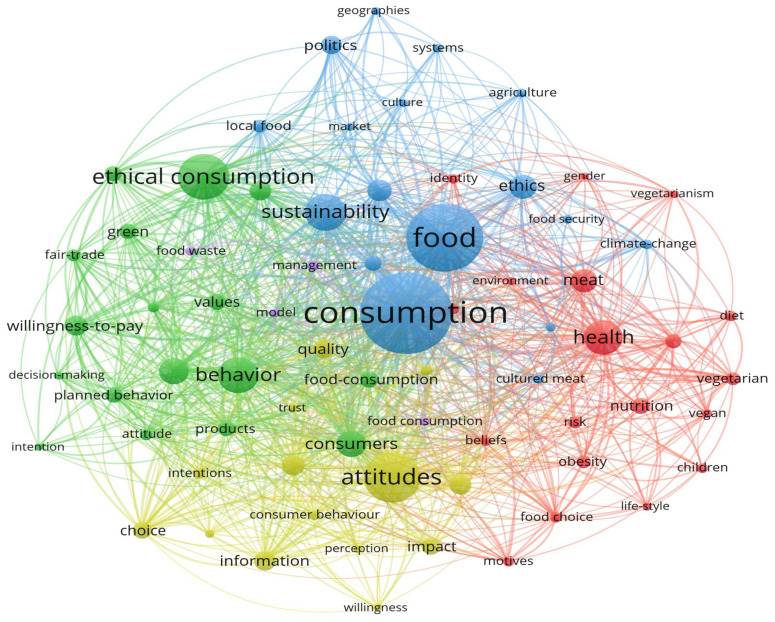
VOSviewer Network visualization of keyword occurrence for words with more than 20 occurrences, N = 72 words meeting the criteria.

Out of a pool of more than 5151 keywords, we identified 72 words that appeared at least 20 times, signaling their significance within the dataset. These keywords were then organized into five distinct clusters (Table 4 and Figure 8) based on their thematic similarities and relationships. This clustering process enables a more focused analysis and interpretation of the extensive dataset, allowing for deeper insights into the various branches of the topic under investigation.

While some authors focused on identifying patterns for the most used keywords in the past [69], others worked on defining methods to help predict future trends [70,71]. Our research followed in their footsteps. Analyzing the trajectory of keyword appearances offers insight into the evolution of academic interests and emerging research areas, and this kind of analysis is particularly valuable for academic institutions, researchers, and policymakers to strategically plan for future developments. It is however essential to reiterate that over 80% of the papers were published within the last decade, making it possible to detect emerging trends by analyzing sudden increases in usage after 2020. By extracting and analyzing the Publication Year and Keywords Plus columns from our data export, we identified the frequency of each keyword by year. Considering keywords that registered a steady increase in mentions over time, such as “ethical consumption” and “sustainability”, we can infer that these areas will continue to attract attention. The growing concern for environmental sustainability and ethical business practices likely means these areas will not only see increased academic focus but also enhanced funding opportunities [72].

**Figure 9:**
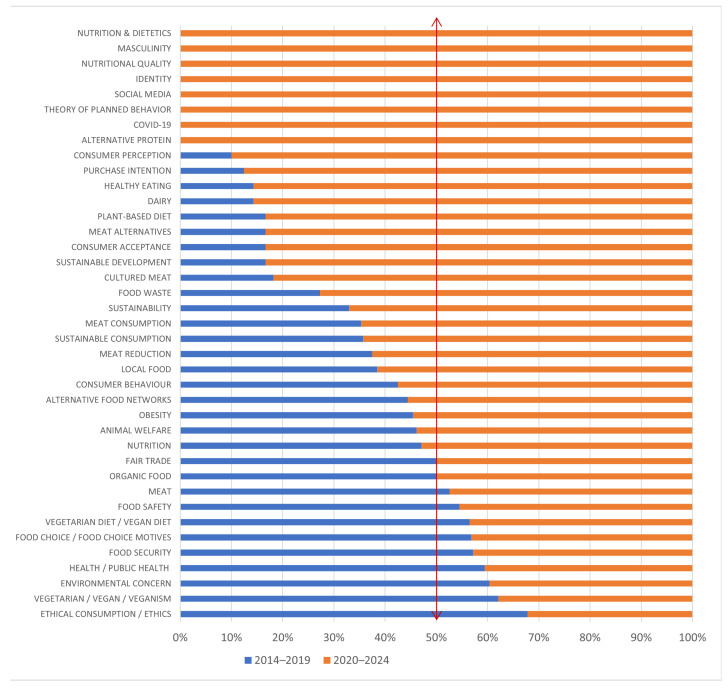
Share of occurrences after 2014 of most popular keywords, as chosen by authors.

Consequently, we observed that certain terms have appeared and have been mentioned exclusively in the past five years (2020–2024). Terms such as “social media”, “theory of planned behavior”, “identity”, “masculinity”, “gen Z”, “alternative proteins”, and related terms tend to occur solely after 2020 (see Figure 9).

As new topics emerge as significant, curriculum developers can integrate these subjects into courses to prepare students for the latest challenges and opportunities in their field. For example, incorporating advanced courses on ethics in technology and business could equip students with the necessary skills to navigate complex ethical landscapes in their professional careers [73]. AI-powered algorithms could optimize food supply chains, reducing waste and promoting sustainability [74], while virtual reality experiences might offer consumers immersive insights into the origins and production methods of their food, fostering greater transparency and ethical awareness [75]. Additionally, robotics could streamline agricultural practices, enhancing efficiency while minimizing environmental impact [76]. As these technologies continue to evolve and integrate into the food system, they are likely to introduce new ethical considerations and reshape the way individuals interact with food, thereby influencing consumption patterns and preferences. So far, very few articles can be found in our dataset that incorporate these emergent technologies. Herrewijn and colleagues [77] explored the benefits of using VR technology to generate empathy for the pigs destined for slaughter, aiming to use technology to reduce meat consumption, Camarena [78] documented the application of AI to enhance sustainability and ethical practices in food production. However, while AI and VR technologies may be more palatable to consumers, Lupton and Turner [79] found that although consumers express interest in ethical and sustainable food options, they are not open to trying more controversial options like 3D-printed food or insect-based food. This suggests that while certain technologies may hold promise for promoting ethical food consumption, consumer acceptance and adoption may vary depending on the perceived palatability and familiarity of the innovations. Further exploration is warranted to understand how emerging technologies can be effectively leveraged to address ethical concerns and reshape consumer behavior in the food domain.

*Q4. What is the relative prevalence of practical approaches versus theoretical models in researching ethical food consumption?*

For this section of our research paper, our attention was directed toward the top 100 papers, chosen based on their citation count, mirroring the analytical approach adopted in our earlier visualization chapters. The breakdown of article types is as follows:

- 79 are classified as articles
- 16 are identified as review papers
- 5 are categorized as articles; proceeding papers

The distribution between theoretical, and conceptual articles and those based on practical approaches is relatively balanced. Approximately 53% of the articles predominantly engage in either quantitative or qualitative research methodologies to investigate issues related to ethical food consumption. This indicates a significant focus on empirical research, encompassing both statistical analysis and in-depth qualitative exploration, aimed at understanding various facets of ethical food consumption. The practical approach in these studies typically involves real-world applications and case studies, which help in grounding theoretical models and conceptual frameworks in observable phenomena. Out of these 53 articles, 18.8% are qualitative studies based on in-depth interviews and focus groups.

These studies primarily seek to identify the barriers that prevent the translation of ethical consumer attitudes into corresponding actions. Real-world limitations play a significant role in shaping actual consumer behavior, often leading to choices that diverge from their ethical intentions [80]. For example, while there is a growing awareness and understanding of the negative impacts of food waste, this awareness does not always translate into actionable behavior change at the individual level [81]. It is also important to note that, although consumers are inclined to make ethical and sustainable choices, their lack of awareness regarding food waste management diminishes their motivation to be ethical consumers [82]. Additionally, the complexity and lack of transparency in the food waste chain can further hinder their ability to make informed and responsible decisions.

The predominant themes in food consumption research—including tools like the Food Choice Questionnaire and its derivatives, as well as topics like organic food consumption, locally produced foods, the attitude–purchase gap, and the willingness to adopt alternative diets—demonstrate an increasing academic interest in unraveling the complexities of consumer behavior and decision-making processes. These subjects are rigorously explored through large-scale quantitative studies designed to provide an eagle-eyed perspective on actual consumer thoughts and actions. Such studies aim to yield statistically significant insights that can inform strategies to influence consumer choices positively. The largest study in our dataset (6189 participants), conducted by Tobler and team in Switzerland in 2010 [83], exemplifies the type of research focused on consumer behavior and its environmental impacts. This study sought to identify which pro-environmental behaviors are most likely to be adopted to mitigate negative environmental effects. Although a majority of respondents acknowledged the necessity of taking action to protect the environment, there was a noticeable reluctance to adopt practices that would significantly alter their food choices. While respondents were generally willing to opt for less processed or packaged foods—a relatively minor adjustment—there was considerable hesitation regarding more substantial changes. Specifically, they showed reluctance to modify their meat consumption habits or to increase their intake of organic foods at the expense of their current diet. This illustrates the complexity of the attitude-behavior gap, where despite recognizing environmental concerns, personal preferences and habitual dietary practices often dominate decision-making processes [84]

Complex studies often involve multiple phases, indicating a shift towards interdisciplinary approaches in research methodology. Initially, researchers conduct in-depth interviews to calibrate their study and develop a model. Subsequently, this model is applied on a larger scale to validate its effectiveness and applicability across broader contexts, highlighting the iterative nature of scientific inquiry and the importance of methodological rigor in advancing knowledge in the field of food consumption studies. This is the case with the paper “Understanding Local Food Shopping: Unpacking the Ethical Dimension” [85], which ran in two phases. Phase 1 involved using focus groups to capture a wide range of perspectives and insights, shedding light on the complexities of consumer preferences and behaviors associated with the support of local food markets. Phase 2 consisted of extending a thoroughly vetted survey to 1223 shoppers. This setup was crucial in assessing how these factors influence both actual and intended purchasing behaviors, providing a structured approach to understanding the dynamics of consumer choices in a quantifiable manner. This would not have been as efficient without Phase 1.

Meanwhile, the conceptual and theoretical articles contribute to developing and refining the underlying concepts and ideas that inform these practical investigations. This complex relationship between theory and practice enriches the academic discourse, offering comprehensive insights into the complexities of ethical food consumption.

The interplay of themes in recent scholarly articles on ethical food consumption demonstrates a complex landscape where environmental concerns [57], technological advancements, consumer behavior, and ethical considerations converge. For instance, the tension between ecological sustainability and economic practicality is evident in discussions about the trade-offs between food production and biofuels, which highlight resource allocation challenges [86]. This intersects with consumer-driven changes in the food market, such as the rising acceptance of cultured meat and plant-based milks, reflecting a shift towards sustainable and ethically produced alternatives. These innovations not only address environmental impacts but also cater to changing dietary preferences and health concerns.

These studies in our dataset underscore the evolving role of consumers who are increasingly wielding their purchasing power to influence food industry practices toward greater ethical and environmental accountability. This shift is further complicated by global challenges like the COVID-19 outbreak, illustrating the need for resilience and adaptability in food production systems [87].

Simultaneously, the concern for animal welfare and ethical treatment in food production connects deeply with consumer decision-making processes, as people become more informed and concerned about the origins and impacts of their food choices. This is paralleled by the exploration of food safety and regulatory frameworks, particularly in the emerging field of edible insects and bio-accessibility assessments, where safety and nutritional validation are crucial [23].

The reviews in the dataset (Table 5) highlight the necessity of an interdisciplinary approach to ethical food consumption and sustainability because combining insights from economics, environmental science, consumer behavior, and public health is crucial to comprehensively address these issues.

Collectively, these themes paint a picture of a sector at the crossroads of tradition and innovation, where the imperative to sustainably feed a growing global population must be balanced with the imperative to do so ethically and responsibly. Each theme not only stands on its merit but also contributes to a broader narrative about the future of food in a rapidly changing world.

*Q5. What emerging ethical issues in the global food chain are likely to gain traction due to increasing consumer empathy, and how can future research address these issues?*

Empathy, as an internal ability to express understanding and share others’ feelings and experiences, means that it can be an important driver in decision-making [95]. Making ethical choices concerning food is a sign of empathy. It drives consumers to consider the welfare of farm animals, the labor conditions of agricultural workers, and the environmental consequences of food production. By fostering a sense of connection and personal responsibility, empathy encourages individuals to make food choices that align with their moral values and contribute to the greater good.

In exploring the role of empathy in ethical food consumption, we conducted a targeted analysis of our database. Specifically, we selected articles featuring “empathy” and related terms, including “emotion”, “compassion”, “sympathy”, “kindness”, and “thoughtfulness”, in their abstracts, author keywords, or Keywords Plus. We intentionally excluded terms such as “concern” and “kind” due to their broader and more ambiguous meanings in academic discourse. This selection process yielded a total of 121 papers published between 2003 and 2024.

**Figure 10:**
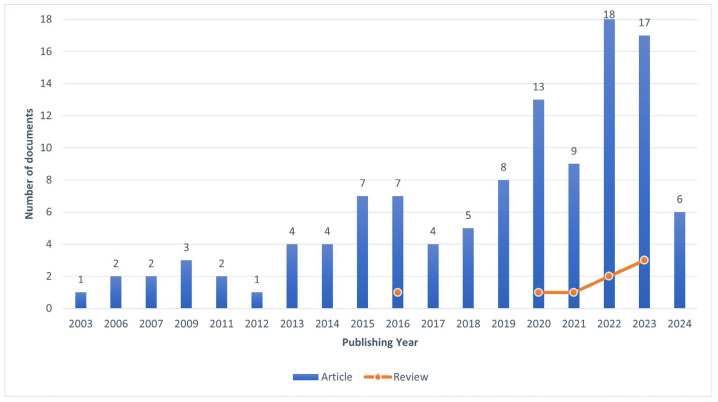
Paper distribution count by Publishing year.

We can identify a slowly rising trend of studies in this area, with more than 80% of the papers being published after 2015. Review papers also appeared after 2015, with the first one being in 2016, counting a total of eight review papers connecting empathy and ethical food consumption (Figure 10).

Following this step, we conducted a word count analysis on the keywords, both as defined by authors and Keyword Plus. Among the top 20 most frequently mentioned keywords, “animal welfare” ranked highest, with 42 occurrences. Other related terms, such as “meat” (19 occurrences) and “meat consumption” (11 occurrences), also appeared in the top 20. This indicates a strong thematic link between empathy and animal welfare. The most referenced paper on this topic [96] relays that the majority of respondents cited animal-related motives for turning to a vegan diet. However, the study also revealed that animal welfare is not the sole reason for choosing a vegan lifestyle; most participants mentioned multiple factors influencing their decision. While health and personal well-being, along with environmental concerns, were frequently cited, no other significant reasons related to empathy were identified. This trend is consistent across the entire list of 122 papers, indicating a notable gap in research exploring other aspects of ethical food consumption in relation to empathy.

**Figure 11:**
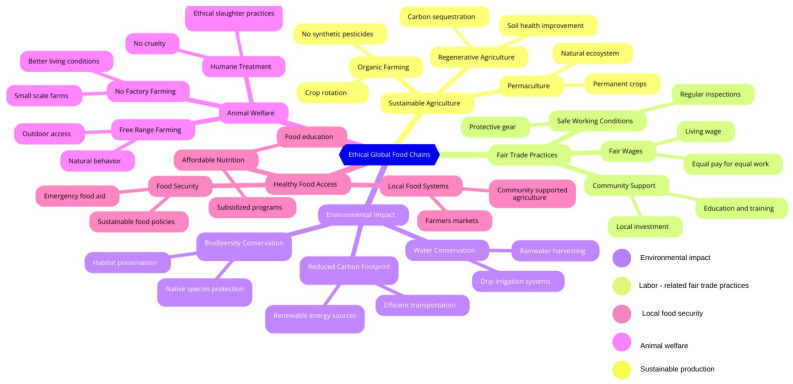
Ethical Global Food Chain—impact and ramifications.

If we map elements of the ethical Global Food Chain [97,98,99,100], we identify additional areas that warrant empathy (Figure 11). Beyond animal welfare, the environment, personal well-being, and several other aspects are deserving of empathetic consideration. These include fair trade practices (concerning workers, working conditions, and gender equality), food security (such as access to resources, community-supported agriculture, affordable food, and sustainable food policies), and broader care for the earth and people (including sustainable production, resource regeneration, and the use of non-harmful substances). Current research, however, does not sufficiently cover these critical areas.

We believe that further focus on this research to include the underrepresented areas is crucial for developing a better understanding of ethical food consumption. Integrating aspects such as fair-trade practices, food security, and sustainable production into the discourse will provide a more holistic view of the global food chain, helping to identify the interconnectedness of these factors and their collective impact on promoting empathy within the food industry. Additionally, by addressing these gaps, future studies can offer more robust solutions and strategies that support not only animal welfare and environmental sustainability but also sustainable production, social equity, and human well-being. Such an inclusive approach is essential for fostering a truly ethical and empathetic global food system.

## 4. Conclusions

Our bibliometric analysis of ethical food consumption research highlights several key trends and insights, offering a detailed overview of the field’s evolution and current state. Additionally, it underscores the critical role of empathy and human values in shaping future research and practices in this domain.

The increasing volume of publications since 2005 reflects a growing scholarly interest in ethical food consumption. This surge aligns with heightened global awareness of the ethical implications associated with food production, distribution, and consumption. However, the concentration of research in developed regions highlights a need for a more inclusive global perspective.

Empathy emerges as a central theme in understanding ethical food consumption. It drives consumers to consider the welfare of farm animals, the labor conditions of agricultural workers, and the environmental impacts of their food choices. By fostering a deeper connection and sense of responsibility, empathy can bridge the gap between ethical intentions and actual consumer behaviors. This “attitude-behavior gap” remains a significant challenge, emphasizing the need for strategies that make ethical choices more accessible and practical for consumers.

Education plays an essential role in promoting ethical food consumption. Equipping consumers with knowledge about food labels, ingredients, sustainability, fair trade, and organic farming empowers them to make informed choices that support environmental stewardship, social equity, and community well-being. Understanding the broader implications of food choices encourages consumers to support agricultural practices that prioritize both human and ecological health.

Emerging technologies, such as artificial intelligence, virtual reality, and robotics, hold promise for transforming the food industry by enhancing supply chain transparency, reducing waste, and enabling informed consumer choices. Integrating ethical considerations with these technological advancements is crucial to ensure they align with human values and sustainability goals. Thorough ethical, ecological, and social evaluations are necessary to harmonize technological progress with the principles of ethical food consumption.

Despite the strong thematic link between empathy and animal welfare, our analysis reveals a notable gap in research exploring other aspects of ethical food consumption in relation to empathy. Future studies should expand to include fair trade practices, food security, and sustainable production, offering a more holistic view of the global food chain. This broader perspective will help identify the interconnectedness of these factors and their collective impact on promoting empathy within the food industry.

In conclusion, fostering a truly ethical and sustainable global food system requires an inclusive approach that integrates empathy and human values into every aspect of food production, distribution, and consumption. By addressing current research gaps and leveraging emerging technologies, we can develop robust solutions and strategies that support not only animal welfare and environmental sustainability but also social equity and human well-being. Such an empathetic and value-driven approach is essential for creating a food system that aligns with our moral principles and supports the greater good.

## Figures and Tables

**Table 1:** Primary information derived from the Web of Science dataset.

Description	Values
Time span	1995–2024
Sources	523
Documents (Articles and Review papers)	1096
Authors	3353
Source Organizations	1441
Countries	92
Cited references	55,317
Cited authors	37,797
Keywords Plus	2396
Author Keywords	3270
Unique keywords	5151

**Table 2:** List of papers on ethical food consumption by the most cited author in the analyzed dataset.

Authors	Article Title	Citations	Year
Vermeir, I.; Verbeke, W. [53]	Sustainable food consumption: Exploring the consumer attitude—behavioral intention gap	1524	2006
Van Loo, E.J.; Caputo, V.; Nayga, R.M.N.; Verbeke, W. [58]	Consumers’ valuation of sustainability labels on meat	235	2014
Verbeke, W.; Vanhonacker, F.; Sioen, I.; Van Camp, J.; De Henauw, S. [59]	Perceived importance of sustainability and ethics related to fish: A consumer behavior perspective	102	2007
Vanhonacker, F.; Verbeke, W. [60]	Buying higher welfare poultry products? Profiling Flemish consumers who do and do not	66	2009
de Graaf, S.; Vanhonacker, F.; Van Loo, E.J.; Bijttebier, J.; Lauwers, L.; Tuyttens, F.A.M.; Verbeke, W. [61]	Market Opportunities for Animal-Friendly Milk in Different Consumer Segments	11	2016
Verbeke, W. [62]	Communicating food and food chain integrity to consumers: lessons from European research	5	2011

**Table 3:** Sources exceeding 500 citations, as per the Web of Science data extraction.

	Source	Documents	Citations
1	*Appetite*	46	4735
2	*Journal of Agricultural and Environmental Ethics*	39	2291
3	*Food Policy*	9	1970
4	*Journal of Business Ethics*	17	1837
5	*Journal of Cleaner Production*	22	1328
6	*International Journal of Consumer Studies*	15	1067
7	*Sustainability*	56	936
8	*Sustainable Development*	2	849
9	*Food Quality and Preference*	25	800
10	*Journal of Consumer Culture*	21	788
11	*British Food Journal*	40	730
12	*Geoforum*	16	578
13	*Journal of Retailing and Consumer Services*	11	535

**Table 4:** Keyword clusters based on occurrence.

Cluster	Words	Focus
1—Red	Beliefs, children, diet, environment, food choice, framework, gender, health, lifestyle, meat, meat-consumption, motives, nutrition, obesity, risk, vegan, vegetarianism, vegetarian	Focus on diverse lifestyle practices, gender dynamics, and dietary preferences encompassing considerations of health, sustainability, and cultural influences
2—Green	Attitude, behavior, consumers, decision-making, ethical consumption, fair trade, green, intention, motivation, organic food, planned behavior, products, sustainable consumption, values, willingness to pay	Focus on the intricate dynamics of consumer attitudes and behaviors towards ethical consumption practices and the growing importance of ethical considerations in shaping purchasing decisions.
3—Blue	Agriculture, animal welfare, challenges, climate change, consumer, consumption, cultured meat, culture, ethics, food, food security, geographies, local food, market, politics, sustainability, system	Focus on sustainable agricultural practices, animal well-being, and climate challenges in the context of ethical food
4—Yellow	Attitudes, choice, consumer behavior, impact, information, intentions, knowledge, perception, perceptions, preferences, quality, trust, willingness	Focus on exploring attitudes and intentions by assessing the impact of information and knowledge on consumer perceptions and preferences
5—Purple	Food consumption, food waste, management, model	Focus on behaviors and habits surrounding food intake, while also addressing strategies for minimizing waste throughout the supply chain.

**Table 5:** Review articles within our dataset.

Source	Article Title	Times Cited (All)	Year
[15]	The citizen-consumer hybrid: ideological tensions and the case of Whole Foods Market	387	2008
[17]	Food Versus Biofuels: Environmental and Economic Costs	297	2009
[21]	Consumer acceptance of cultured meat: A systematic review	250	2018
[36]	A discussion paper on challenges and limitations to water reuse and hygiene in the food industry	190	2005
[22]	Plant-based Milks: A Review of the Science Underpinning Their Design, Fabrication, and Performance	170	2019
[38]	Are dietary intakes and eating behaviors related to childhood obesity? A comprehensive review of the evidence	170	2007
[88]	Ethically minded consumer behavior: Scale review, development, and validation	168	2016
[23]	Edible insects: An overview on nutritional characteristics, safety, farming, production technologies, regulatory framework, and socio-economic and ethical implications	157	2020
[87]	Challenges to the Poultry Industry: Current Perspectives and Strategic Future After the COVID-19 Outbreak	156	2020
[89]	China’s energy status: A critical look at fossils and renewable options	150	2018
[90]	What We Know about the Public’s Level of Concern for Farm Animal Welfare in Food Production in Developed Countries	147	2016
[91]	Bioaccessibility assessment methodologies and their consequences for the risk-benefit evaluation of food	141	2015
[92]	Linking sustainable product attributes and consumer decision-making: Insights from a systematic review	130	2020
[93]	A Comprehensive Review of the Benefits of and the Barriers to the Switch to a Plant-Based Diet	109	2020
[94]	Application of the Food Choice Questionnaire across cultures: Systematic review of cross-cultural and single country studies	95	2018
[86]	Biofuel Impacts on World Food Supply: Use of Fossil Fuel, Land and Water Resources	95	2008

## Data Availability

No new data were created or analyzed in this study.
